# “We want it to be a culture”: children and young people’s perceptions of what underpins and undermines education-based wellbeing provision

**DOI:** 10.1186/s12889-023-15836-z

**Published:** 2023-07-07

**Authors:** Ola Demkowicz, Kirsty Pert, Caroline Bond, Emma Ashworth, Alexandra Hennessey, Lucy Bray

**Affiliations:** 1 Manchester Institute of Education, The University of Manchester, Manchester, UK; 2 School of Psychology, Liverpool John Moores University, Liverpool, UK; 3 Nursing and Midwifery, Edge Hill University, Ormskirk, UK

**Keywords:** Children and young people, Child and youth voice, Wellbeing, Education, School-based wellbeing provision, Qualitative

## Abstract

**Background:**

Provision that aims to promote the social, emotional, and mental wellbeing of children and young people (including their mental health) is increasingly implemented in education settings. As researchers, policymakers, and practitioners explore the complexities of promotion and prevention provision in practice, it is critical that we include and amplify children and young people’s perspectives. In the current study, we explore children and young people’s perceptions of the values, conditions, and foundations that *underpin* effective social, emotional, and mental wellbeing provision.

**Methods:**

We engaged in remote focus groups with 49 children and young people aged 6–17 years across diverse settings and backgrounds, using a storybook in which participants constructed wellbeing provision for a fictional setting.

**Analysis:**

Using reflexive thematic analysis, we constructed six main themes presenting participants’ perceptions: (1) recognising and facilitating the setting as a caring social community; (2) enabling wellbeing to be a central setting priority; (3) facilitating strong relationships with staff who understand and care about wellbeing; (4) engaging children and young people as active partners; (5) adapting to collective and individual needs; and (6) being discreet and sensitive to vulnerability.

**Conclusions:**

Our analysis presents a vision from children and young people of an integrated systems approach to wellbeing provision, with a relational, participatory culture in which wellbeing and student needs are prioritised. However, our participants identified a range of tensions that risk *undermining* efforts to promote wellbeing. Achieving children and young people’s vision for an integrated culture of wellbeing will require critical reflection and change to address the current challenges faced by education settings, systems, and staff.

**Supplementary Information:**

The online version contains supplementary material available at 10.1186/s12889-023-15836-z.

## Background

National and international public health policy increasingly foregrounds education settings as sites for promotion and prevention in children and young people’s social, emotional, and mental wellbeing.^1^ The World Health Organization regularly recommends that schools promote health, including wellbeing, and recently published global standards and indicators for wellbeing provision in schools [1]. In England, a 2017 Green Paper presented a view to ‘transform’ mental health provision, with considerable emphasis on the role of education settings [2]. Yet, there is a paucity of research exploring children and young people’s perceptions of how wellbeing and mental health provision should be delivered within the school setting. We set out to address this gap within this paper.

Children and young people spend a significant proportion of their time in schools and colleges, in principle creating an ideal environment to embed wellbeing promotion in daily life and respond to emergent issues, such as early signs of mental health difficulties. Within such provision, there is an emphasis on integrated delivery of several approaches for best effect [3]: *whole school approaches*, a multilevel approach that embeds and connects varied provision across the culture and stakeholders [4]; *universal provision*, designed to foster skills among *all*, often through taught content; e.g., emotion regulation, coping, social skills [5]; and *targeted provision*, offering additional support or skill-building for those at increased risk of poorer outcomes [6].

Wellbeing provision does not occur in a vacuum, but within the complex adaptive system of each individual education setting, which itself sits within wider local, regional, national, and international contexts. That is, the conditions of each educational setting provide a backdrop that can affect provision delivery and impact [7]. At the individual setting level, for instance, *school climate* reflects features such as norms, values, relationships, teaching and learning practices, and organisational aspects [8]; in a *positive* school climate individuals would feel safe physically, socially, and emotionally. More widely, at a national level, there are tensions in how provision sits within policy and infrastructure, including under-resourcing (financially and in training/support), growing demands on a stretched workforce, and a socio-political landscape systematically heightening wellbeing needs [9–11]. These complexities remain under-researched, and ever-evolving policy and societal landscapes necessitate ongoing investigation.

It is critical that we amplify the voices of children and young people as central stakeholders whose views are often overlooked, or indeed not elicited in the first place. It is promising that there is increasing recognition of the voice of children and young people in public health policy and practice, and a growing body of research is beginning to offer valuable insights into their views on wellbeing provision in education. Yet, these are often centred around evaluations of specific interventions, to highlight how context influences children and young people’s engagement with particular wellbeing models and approaches, as opposed to wellbeing provision generally. One example of such a finding is the issue of vulnerability and safety in the classroom setting; McKeague et al. [12] report that young people in their study felt that the classroom provided a safe, familiar space for an intervention targeting emotional difficulties, while Hailwood [13] conversely described young people feeling unsafe closing their eyes during mindfulness exercises, given the presence of classmates. Beyond interventions, some studies have explored more embedded aspects such as staff support; Spencer et al. [14] and Stapley et al. [15] describe how young people reflect on how *some* teachers are seen as able to provide valuable early support, but in general question teachers’ responsiveness, availability, and knowledge. Very few studies have explored more general and wider perceptions of provision beyond specific interventions and aspects of provision, though some such work has taken place and offered valuable insights, particularly around child and youth voice. Simmons et al. [16] emphasised the need for students to have a say in how wellbeing provision is delivered and works, while Atkinson et al. [17] reported on a co-produced school mental health strategy and emphasised their students’ desire to be involved in decisions. Taken together, such studies offer *some* insights, including how peers form part of the landscape of provision, complexities in how teachers are – and are not – viewed as support routes, and the importance of children and young people being involved in decision-making.

In general, children and young people’s views on wellbeing provision in educational settings are under-represented in the evidence, despite their participation having the potential to meaningfully develop and enhance provision [16]. Existing studies are often limited by specificity to developmental stages, educational setting types, and aspects of provision; this would be complemented by exploration that goes beyond specific contexts, groups, and practices to progress ‘big picture’ policy and provision. There are also methodological limitations including frequent failure to engage in consultation with children and young people as part of the research process, which can greatly enhance methods and in turn the relevance and meaningfulness of research [18].

### Current study

We narrow critical gaps here by exploring children and young people’s perceptions of the values, conditions, and foundations that *underpin* effective social, emotional, and mental wellbeing provision within an educational setting. Thus, this study draws on focus group data generated through a project funded by The National Institute for Health and Care Excellence (NICE),^2^ where we used creative and inclusive methods to speak with children and young people aged 6 to 17 years from varied education setting types and backgrounds. Use of this data offers several strengths. First, we included participants from a broad age range and varying educational and demographic characteristics, including those often seldom heard in research, to ensure that our analysis provides a wide perspective offering significant policy and practice implications. Second, we engaged in ‘ideal world’ discussions with children and young people to explore what *they* felt was important in promoting wellbeing, and so our exploration of these issues reflects children and young people’s aspirational views s rather than only a reflection on specific practices and pockets of experience, allowing an examination of how things could work rather than only how they do work. Third, we adopted methodological best practices including consulting with children and young people as research advisors, and open research practices by sharing our data generation materials [20] and reflections [21], and analytic codes (see Supplementary Materials). Together, such approaches situate the study as a rigorous, original contribution to knowledge and understanding of how the context of the education setting provides a nuanced basis for provision as a whole, beyond specific strategies and domains. Greater understanding, particularly driven by children and young people’s views, can shape public health policy and practice to best deliver wellbeing promotion and prevention provision in educational spaces.

## Methods

### Research design and team

We adopted a qualitative design to elicit rich, detailed insight. We engaged diverse participants across age groups, setting types, and demographic backgrounds, including those not always ‘heard’ in research (e.g., in alternative^3^ and special educational provision; from low-income families, UK ethnic minority groups). We undertook remote online focus groups using a storybook that asked participants to design wellbeing provision in a fictional school setting. These focus groups occurred in May to July 2021, when education settings in England opened to all pupils after disruptions in the COVID-19 pandemic, and so we conducted focus groups remotely given social distancing restrictions at the time. We engaged in public involvement and engagement consultation with 10 children and young people in two groups (primary- and secondary-aged, respectively). This online consultation informed the development of our creative methods, focused the activities on general perceptions rather than personal experience, guided approaches to remote engagement, and shaped decisions around staff presence during discussions. For a detailed account and reflection on our design, see Hennessey et al. [21].

We adopt a social constructionist epistemological lens, wherein reality is socially constructed, recognising that participants’ perspectives have been constructed in a social setting, their discussions took place in a group context, and our interpretation of these discussions is grounded in our own experiences. With that in mind, we describe our team: we are researchers interested in how education and other services can provide effective, appropriate wellbeing support, with expertise in qualitative inquiry with children and young people. Authors 1 (OD) and 5 (AH) jointly led the main research project funded by NICE, Author 2 (KP) led focus groups (with an assistant; see acknowledgements), Authors 4 (EA) and 6 (LB) collaborated on the main project, and Author 3 (CB) joined for this paper. We have wider interests that perhaps influenced our approach and interpretation, including in specific intervention types, developmental psychopathology, healthcare, and special educational needs and disability (SEND) provision. KP has experience as a secondary school teacher, and CB has worked as a primary school teacher and an Educational Psychologist supporting secondary schools with wellbeing provision. Authors KP and LB are parents to children and young people of school age.

### Sampling and participants

We engaged 49 children and young people across seven focus groups, each with five to eight participants. This sample size, number of groups, and group size are moderate for focus group research [22], decided upon to balance our emphasis on engaging a variety of perspectives with our focus on rich, in-depth discussion of perceptions of a specific area of participants’ lives. After consultation with settings, we constructed smaller groups for participants who were younger or in special provision, to facilitate their engagement and be responsive to their needs.

We recruited via education settings, advertising through local and national networks (e.g., Schools in Mind, Research Schools Network, Twitter). From interested settings, we used purposive maximum variation sampling to identify diverse settings, across English education phases (‘key stages’), setting types (mainstream, special, and alternative provision), geographic regions across England, and setting-level demographics [23] to select seven settings with varied proportions of cohorts eligible for free school meals (FSM), speaking English as an additional language (EAL), and receiving SEND support.

We engaged with setting staff as gatekeepers to facilitate inviting participants, emphasising g in our discussions with them our focus on including seldom heard voices. In each setting/group, participants were in the same class (year group in mainstream, or general grouping in special and alternative provision, which is not always based on tight age brackets). Our participants were aged 6 to 17 years, across Key Stages 1 to 4 and post-16 provision (English education stages), and 10 were in special or alternative provision. 49% (n = 23) were boys, 40% (n = 20) were eligible for FSM, 23% (n = 11) were identified as having SEND, 32% (n = 15) spoke EAL, and 61% (n = 29) were of White British ethnicity^4^. These figures deviate from national norms, showing as intended greater proportions of FSM eligibility, SEND, EAL, and non-White ethnicity than the general child and youth population in England [24].

### Data generation

We used focus groups, rather than one-to-one interviews, for contrast, challenge, clarification, and ownership of ideas [25]. These were remote and online, with participants in each group together within the educational setting space with a member of staff, and two researchers joining via videoconferencing software (Zoom or Microsoft Teams, by setting preference). We gave guidance to education staff on supporting the groups, and those staff members present signed confidentiality agreements. Focus groups lasted at most one hour to limit fatigue and burden. Following consultation with children and young people, we created a ‘storybook’ with images, visual prompts, and vignettes, inviting participants to imagine themselves as headteachers and make leadership decisions. Creative approaches and vignettes are advised for engagement with children and young people [26], and our storybook helped participants explore higher-level ideas (e.g., whole school system and climate) about provision without requiring them to divulge or discuss personal experiences. Our storybooks, schedules, and creative resources are freely available via the Open Science Framework (OSF) [20]. We sent storybooks, and materials for primary-aged participants (e.g., a ‘school’ colouring picture, pens, headteacher name stickers, and ‘lightbulb lollipops’ to hold up for turn-taking). Storybooks explored varied wellbeing approaches: whole school, universal, targeted, and (as of particular interest to NICE), transition support. The storybooks and associated discussion prompted open-ended concepts to be explored inductively, rather than imposing closed questions. For instance, a section on targeted provision for primary schools, “Helping Children”, introduced vignettes about children who may benefit from specific support: “Sunny in Class One is finding it hard to play with other children. Sunny might need some help learning how to make friends.” We facilitated participants in collectively discussing this and other similar vignettes, offering questions including whether it was important for the setting to help, how to know who needed help, who staff should consult with when deciding how to help, and what help might entail.

We recorded discussions via videoconferencing software and transcribed them verbatim. Demographic information was provided by a parent/carer or teacher for those in primary education, and participants themselves in secondary and post-16 settings. To recognise contributions, we provided a £10 voucher and “Active Citizenship” certificate.

### Ethical considerations

The study received ethical approval from The University of Manchester Research Ethics Committee (ref. 2021-11252-19677). We used opt-in consent/assent, and conveyed information via multiple methods in line with guidance [27], providing information sheets for children and young people and, for those in primary and secondary education, a parent/carer information sheet and consent form. Interested students were shown a video with key information about the project and short ‘talking heads’ to introduce the children and young people to the staff who would lead the focus groups. At focus groups, researchers reiterated information, addressed questions, and established assent or (in post-16 provision) consent. We provided signposting, and setting staff were available in case of any children and young people becoming upset. Setting staff signed confidentiality agreements and we used storybooks to ensure participants were not asked to disclose personal experiences and so could talk freely in front of setting staff.

### Data analysis process

We analysed data using Braun and Clarke’s [28] six-stage reflexive thematic analysis. This is in line with our social constructionist lens, and offered value as it is used to explore patterns across perceptions as well as richness and nuance within and across accounts [28]. This approach inherently recognises the role of the researcher as actively constructing interpretations [28, 29], and can be carried out with a ‘latent’ lens, which goes beyond descriptive accounts of explicit statements and explores possible underlying meanings and ideas that may have shaped them [28]. This aligns with our emphasis in this analysis on going beyond specific approaches and instead exploring what children and young people perceive to be the values, conditions, and foundations needed to underpin provision, which may be both explicitly *and* implicitly stated.

Analysis was undertaken by Author 1 (OD), facilitated in NVivo Version 12, and supported through reflexive debriefing with the Author 2 (KP). First, OD read and re-read accounts for familiarity. Second, OD systematically coded transcripts line-by-line, identifying and making notes on units relevant to our aim with semantic coding (descriptive of explicit statements) *and* latent coding (exploring possible underlying meanings). Third, OD reviewed coding across transcripts to begin combining codes to construct themes. Fourth, OD reviewed and refined themes, revisiting data in each and exploring the overall thematic structure against the full dataset, and then defined and named themes. Here, KP reviewed the NVivo file alongside OD’s overview of themes, and OD and KP engaged in reflexive discussion and further refinement. Finally, OD constructed a written narrative. Supplementary Materials include a list of codes underpinning our themes.

Our analysis is not a definitive reading of this data. Reflexive thematic analysis is interpretative, with researchers constructing themes. Rather, this is *one* credible account of how our participants viewed the underpinnings of education-based wellbeing provision. We sought to engage with data rigorously and carefully, drawing on guidance on reflexivity and trustworthiness [29,30].

## Analysis

**Fig. 1:**
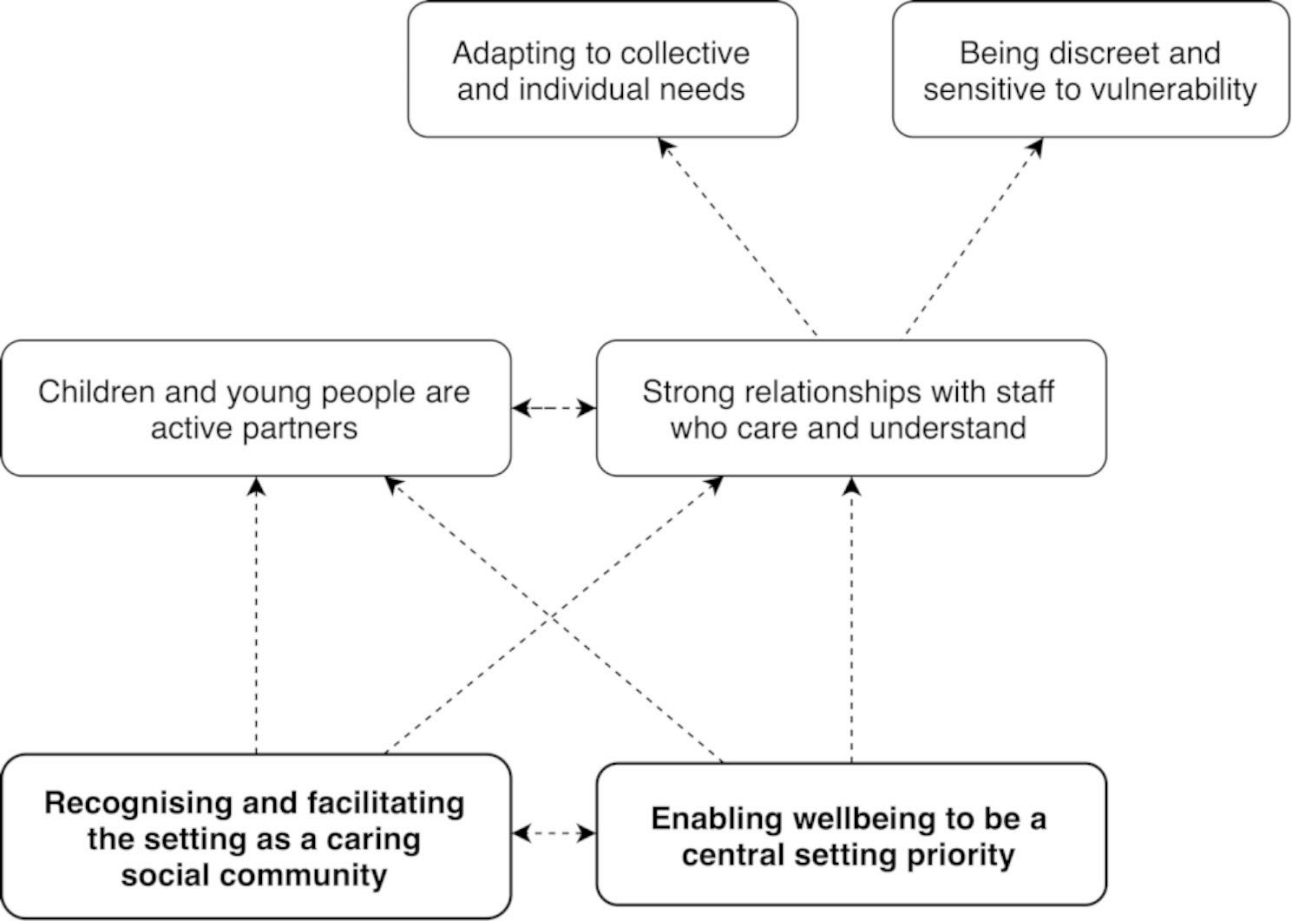
*Study themes, shown as a structure.* We conceptualise those components at the lower level(s) as providing foundational context for each subsequent layer, and thus present these in bold

We constructed six main themes: (1) *recognising and facilitating the setting as a caring social community; (2) enabling wellbeing to be a central setting priority; (3) facilitating strong relationships with staff who understand and care about wellbeing*; (4) *engaging children and young people as active partners; (5) adapting to collective and individual needs; and (6) being discreet and sensitive to vulnerability.* We propose that the components captured in these themes can be seen as existing in ‘levels’, with each level providing foundational context for subsequent levels, as shown in Fig. 1. That is, effectively building a social community where wellbeing is a central priority provides the basis for a setting in which children and young people are active partners in provision, and where staff can have strong relationships with students and care about their wellbeing. In turn, staff can adapt to collective and individual needs and be discreet and sensitive. We do not propose this as a hierarchy; instead, we present levels as inter-related, with each providing context for – rather than being more important than – those at subsequent levels. We note also here that although our aim focused on what under*pins* provision, participants also explored aspects that under*mine* provision, and we explore these in our write-up.

In offering quotations, we note the provision and key stage of the participant. This is offered as context rather than intending to indicate that a theme more heavily reflects any one age group or provision type; where this is the case, we specifically note this. Our use of quotations shows greater coverage of older participants’ input, but this is only as the points they offered tended to be more articulate and succinct for the purposes of illustrating key points. Table 1 clarifies the age groups of key stages within the English education system.

**Table 1:** Key Stage Corresponding to Age Ranges in England

Key Stage	Age Range
Key Stage 1 (KS1)	5–7 years
Key Stage 2 (KS2)	7–11 years
Key Stage 3 (KS3)	11–14 years
Key Stage 4 (KS4)	14–16 years
Post-16 provision	Varied; compulsory education in England is 16–18 years and we focused on this group, but many post-16 provision options extend to age 25

### Recognising and facilitating the setting as a caring social community

In their fictional setting, participants emphasised a caring culture, driven by kindness and compassion from adults *and* students, exploring concepts such as “kind”, “friendly”, “caring” and “compassionate”, “respect”, around setting culture, classroom culture, and individualised support. They presented this as a fundamental premise of wellbeing efforts and felt that this could support social harmony: “because people need to be kind to each other. That’s how it works. We live together” (mainstream KS4 participant); “so people are not horrible to others and they don’t get into fights” (special school KS2 participant). They indicated high importance for these relational foundations, more than features such as conduct rules:

*I think there’s particular [values] that are more important than others.... like just generally just being nice to people and things like that I think those ones are more important like values or rules but like, I don’t know, you know the ones you have in secondary school your like uniform and walk on the left side of the corridor.*

*Researcher: okay so what kind of things would you have then?*

*Erm, just like general things just respect for the people around you.*

(mainstream post-16 participant)

Participants described settings as social spaces at their core, and felt this should be fostered; encouraging cohesion, facilitating friendships, reducing bullying, and teaching social skills, “I think that if you can have people being friends with each other they won’t be that much bullying from each other” (mainstream KS1 participant). Some felt that one *could* teach social skills in a curriculum-based approach, but this alone might not translate to real life: “I don’t think that anyone’s going to leave a lesson about how to build friends and go and make lots of new friends. I don’t I don’t think they would be that effective” (mainstream KS4 participant). Nevertheless, participants suggested social learning and fostering relationships more generally could avoid consequences such as loneliness or mental health difficulties: “kids like that who have no one to speak to and have no have no friends get very depressed” (alternative provision KS4 participant). Participants felt time to reconnect after COVID-19 school closures was important, as this disruption had been a loss: “not getting on with your work, maybe let them chat to other people for a while ‘cause you’ve not seen each other” (alternative provision KS4 participant).

### Enabling wellbeing to be a central setting priority

Beyond the social community context, participants felt that promoting and supporting wellbeing more generally was an important function of education settings, including being attentive to wellbeing, creating time to talk about wellbeing, providing support in difficult times, and early intervention. Younger participants talked about fostering a general atmosphere of staff being caring and supportive, and older participants spoke of responsibility around wellbeing on the part of teachers and settings; that is, something settings ‘ought’ to do:

*Teachers are there like to teach but also to look after the kids […] the teachers will take some responsibility, make sure that everyone in the class is at least looking fine and looking happy* (mainstream post-16 participant).

Participants emphasised a need for a *genuinely* embedded culture of wellbeing; a younger group came up with wellbeing-oriented names for their fictional setting, including “The Mental Health School” and “The Calm School” (mainstream KS1 participants), and participants talked of a sustained, consistent approach: “we don’t want it to be a rule we want it to be a culture and how is culture made? It’s made by time so as the time passes” (mainstream KS4 participant). Participants pointed to the importance of creating sufficient space and time for provision; for instance, on universal taught sessions, “I don’t think that it should be like part of a lesson I think it should only be a whole lesson so that would be like more time” (mainstream KS3 participant). Others talked about how regular engagement could normalise wellbeing discussions: “if you do lots of these things regularly, obviously fitting it around the normal school curriculum that will help normalise it” (mainstream post-16 participant). To create a culture where wellbeing plays a central role, participants emphasised that *everyone* needs to buy in, “regardless of authority”; otherwise, there would be an “epic divide and the values won’t even matter” (mainstream post-16 participant).

Although participants agreed that settings could promote wellbeing *in theory*, in practice contradictory considerations of competing agendas, wider pressures, and questions of responsibility lingered. Some did not feel wellbeing support was the responsibility of teachers, and for instance should be reserved for pastoral staff: “it’s [a teacher’s] job to teach us but it’s not their job to tell us how to feel, tell us what emotions are and how to control them”. Some pointed to demands like exams and, more recently, pressure to ‘catch up’ after school closures. Through this lens, efforts to promote wellbeing appear frustratingly hypocritical:

*You’re saying that that we should make lessons for to address mental health but why is that it’s because of school mostly, because at school you have exams and, I know that’s just how life is but you’re causing it and you’re just trying to fix it, isn’t it? […] Come on!* (mainstream KS4 participant)

Some highlighted a need for higher-level decision-makers, such as Government or setting governors, to address inconsistences, lack of resources, and competing agendas in settings:

*The school can’t really do anything about it but maybe get the governors to try and reduce content because we’ve all missed a lot of learning anyway and there’s the pressure dealing with all the sort of anxiety and stress and a lot of these issues aren’t going to be resolved without more funding allocated to the school for mental health and I don’t really know how the government is able like will be able to do that* (mainstream post-16 participant)

### Facilitating strong relationships with staff who understand and care about wellbeing

Participants highlighted the need for a warm staff persona, particularly among teachers, pointing to relational dynamics including staff being genuine and human, creating comfort and safety, offering mutual respect and flexibility, and being trustworthy:

*Obviously they have to follow the rules but just like robotically just like ‘yeah this this’ straight out the text book rather than trying to build the relationship, you know, up to their discretion or […] behavioural management is handed out and things like that so like if you have a good relationship with your teacher and you trust them and they give some genuine advice to you* (mainstream KS4 participant)

Participants often pointed to the trust that can develop with familiar teachers, which they suggested made teachers preferable over pastoral or external staff for wellbeing provision:

*If some like randomer... comes from outside and like even though they’re a specialist I’m not going to start telling them everything that’s going on I’m going to want someone who I’ve got already got a relationship with* (alternative provision KS4 participant)

They felt that this familiarity and daily interaction meant staff could check in regularly and notice “signs” of difficulties:

*Just noticing certain things like the if they the way they behave in school, if they’re quiet and like extremely quiet could be a sign that there is something going on […] lots of teachers at my school especially my form teacher, as we left on the morning of school did ask if we were okay and if we had anything to say could stay back for five minutes and tell him* (mainstream post-16 participant).

Alongside this, participants indicated it was important that settings encourage and facilitate children and young people in sharing their feelings and asking for help: “we want the children to know that they can speak to anyone at any time” (mainstream KS2 participant).

Participants pointed to challenges here, reflecting that in reality, students might not want to speak to teachers about wellbeing, preferring to discuss this with peers. Some discussed an impression that teachers did not genuinely care about or understand wellbeing, and some emphasised hierarchical power dynamics and an emphasis on being strict and disciplinary as counterintuitive to discussing wellbeing issues. Participants in alternative provision were especially firm here, suggesting “nobody” would seek help from teachers as “teachers are seen as more of a, in the nicest way possible, pest” and “if you say something to them it’s going to get taken the wrong way” (alternative provision KS4 participant).

Children and young people pointed to a need for improved staff support and training to ensure that staff are “qualified” (special school KS2 participant), and thus understand and can talk about wellbeing effectively, and cope with the emotional demands:

*I do think that we need an extra, maybe just like a lesson on the teacher training days on what to say what not to say and not to lump the students together and label them as a mental health illness or dismiss them* (mainstream post-16 participant)

### Engaging children and young people as active partners in wellbeing provision

Participants were clear that staff *and* children and young people should be part of decisions: “students got to have a say as well and obviously staff are grownups” (alternative provision KS4 participant). Older participants emphasised this should be inclusive, with wide consultation such as surveying everyone and setting up panels to identify next steps:

*Everyone should somewhat have a say […] obviously there’ll be things that you don’t agree with that other people do but you need to come up with like a medium ground where everyone somewhat agrees* (mainstream KS4 participant).

Participants often indicated that children and young people can play a role for one another in provision; this included roles for friends, peers more generally, and older students within a setting. Participants felt that peers are seen as more trustworthy: “with the kids you say something to them and they think like you ‘cause they’re a kid as well” (alternative provision KS4 participant), and participants suggested that settings could (sensitively) create space for peers to talk together (e.g., in taught lessons on wellbeing, they could discuss their thoughts in smaller groups): “so it’s not a teacher in lesson it’s groups of people talking to each other because students are going to listen to students more than listen to a teacher and that really students also help each other without even trying” (mainstream post-16 participant). Participants suggested that connecting children and young people with friends or buddies to help them during difficulties or transitions (or encouraging existing friends to support): “we could go over to [a child in the storybook] and say ‘I’ve seen someone that’s a bit sad, do you want to go see him maybe you can make friends?’” (mainstream KS1 participant).

### Adapting to collective and individual needs

Participants considered how provision in their fictional settings could match students’ needs. For instance, with educational transitions and returning after school closures, they explored how settings could make them feel comfortable and safe: “[don’t] throw them into like the whole routine […] try and slowly introduce them and let them know there is support” (alternative provision KS4 participant). They emphasised flexible, personalised approaches to adapt to individual needs:

*The school isn’t just like for one place there’s a range of people here who all have different needs and will all want maybe something different and it’s important to get that kind of inclusion from all of them* (mainstream post-16 participant).

Examples here included allowing children and young people time out as needed, identifying and addressing barriers in the classroom, and considering when discipline might (and might not) be appropriate: “just say like kid with ADHD [attention deficit hyperactivity disorder] who’s kicking off, give him time to be on his own instead of just putting him straight in detention” (mainstream KS3 participant).

### Being discreet and sensitive to vulnerability

Participants highlighted sensitivity to vulnerability around wellbeing, particularly for those experiencing difficulties. Participants suggested these individuals may feel vulnerable or stigmatised in class-based wellbeing discussions^5^: “if other people don’t have the same feelings as them they might feel like they’re not they’re like more alone and they’re not normal things” (mainstream post-16 participant). Participants raised concern around individualised support; first, that this may be stigmatising, leading to judgement and mockery: “they’d probably be like make jokes you know, say, because of what their problem is ‘ah yeah you you you need help’ or ‘you can’t control yourself’ or ‘you can’t make friends’” (mainstream KS4 participant); second, that this could appear unequal and prompt jealousy: “other kids might turn out jealous that [children are] getting all this extra help” (mainstream KS2 participant). Thus, participants suggested creating opportunities for discreet one-to-one disclosures or help-seeking, that peers are not made aware when support is in place (including discreet ways of attending wellbeing/pastoral spaces), and that stigmatising behaviour is challenged: “lots of people used to take the mick out of people who went in [a wellbeing space in a previous school] which was awful and they didn’t get told off for it. They didn’t stop that” (mainstream post-16 participant).

Participants talked often about confidentiality, without fear of information sharing, but felt this was not always the case. They knew teachers are sometimes *required* to share: “we trust a lot of kids more than adults because as adults in a school you know that whatever you say is going to be passed onto someone else if it gets bad” (alternative provision KS4 participant), but also noted a risk of informal sharing:

*How do you know that it’s actually confidential? Like within a claim you claim, er, that teacher can go home, say to their husband or wife like ‘ah this this this happened today. Just don’t tell anybody because it’s supposed to be kept confidential’. […] A teacher could tell another teacher and another teacher but it would all just be kept confidential between each other* (mainstream post-16 participant).

## Discussion

Our analysis offers insights into the values, conditions, and foundations that children and young people perceive to be underpinning effective social, emotional, and mental wellbeing provision in education settings. We present a vision from children and young people of an integrated approach, with a relational and participatory culture. These discussions reflect to some extent an aspirational ‘ideal world’ view of how wellbeing *could* best fit within education settings, but also point to ‘real world’ tensions that children and young people experience. In this sense, we found ourselves presenting not only the values, conditions, foundations that under*pin* provision, but also, critically, those that can serve to under*mine* it.

### Culture and ethos

This study offers critical insight into how children and young people conceptualise the embedded nature of wellbeing provision in education settings, and the way that a setting’s culture provides a foundation for, and even forms part of, wellbeing provision. Participants strongly endorsed an overarching culture that goes beyond discrete social and emotional learning lessons or focused elements of support for those experiencing difficulties, and instead positioned wellbeing and social connection as a core component of educational life. With many existing qualitative studies focusing on specific interventions, this is an important advancement in conceptualising children and young people’s views on how wellbeing provision *should be* implemented in education. Participants focused on the need to actively facilitate a social community where there are trusted key adults and peers able to provide support. As has been discussed elsewhere, participants recognised that creating and facilitating such a culture takes time [31] and investment, such as training for staff and priority-setting [32]. They also recognised that settings would need to be flexible and responsive to pupil needs, both generally and in the return to settings post COVID-19, which has been identified elsewhere by young people and teachers as key in the aftermath of school closures [33,34]. Although our participants did not explicitly discuss the role of senior leadership, their emphasis on an integrated approach highlights the need for leaders to be actively involved ‘on the ground’ in facilitating a positive culture around wellbeing [32]. This reflects a recent qualitative study with UK secondary school students, staff, and parents, where leadership engagement and promotion of a supportive wellbeing-focused culture was emphasised [35]. Furthermore, they emphasised that children and young people themselves should have a voice and active role in provision. Taken together, our analysis echoes wider research supporting a relational approach to education-based wellbeing provision, recognising the complex social systems that influence children and young people collectively and as individuals [36], and advances knowledge and understanding of the ways in which children and young people themselves engage with these complex influences.

Facilitating such a culture is not without challenges, and our analysis shows that contradictions and tensions in discourses and practices do not go unnoticed. Participants pointed to wellbeing and learning agendas as competing with and contradicting one another, and questioned whether wellbeing is a *genuine* function of education. These issues risk undermining trust and encouraging scepticism regarding wellbeing provision and the authenticity of such efforts, especially over time as children and young people deal with more complex wellbeing issues, face increased academic demand, and become better able to observe systemic contradictions. Our focus groups occurred soon after school closures in England, perhaps bringing such tensions to the fore, but these are not new issues, with longstanding concerns that education settings are expected to shoehorn wellbeing provision into a stretched system [9–11]. Our study critically demonstrates that these issues risk affecting how children and young people view and engage with provision; such stark commentary from them on perceptions of a mismatch here has not to our knowledge been observed in other research. Thus, the crux of our analysis is the need for an authentic culture of wellbeing that goes beyond tokenism, including meaningful participatory engagement with students and effective resourcing and training to support staff. If we continue positioning education for learning *and* wellbeing, policymakers, practitioners, and academics must develop innovative ways to integrate these agendas as complementary, and not competing. This requires critical systematic change, including perhaps a move away from traditional practices; for instance, such shifts could include reconsideration of high-pressure ‘single assessment’ approaches to high stakes exams [37], challenging neoliberal agendas that create demand for education settings (e.g., through intense accountability expectations [38]), and effective resourcing to support wellbeing provision [9].

Our analysis highlights that provision ought to begin at the whole system level, not only through discrete interventions but through policy and practice embedded into the daily life of the setting. Despite the ubiquity of wellbeing interventions in education systems, UK education-based evaluations s of such provision often show limited or null effects (e.g., [39–41]). Our analysis, indicating that wellbeing support needs to be flexible, long-term, integrated into the culture, embraced by teachers, and informed by pupils’ voices, may help to explain this. Interventions are often highly prescriptive, with a limited set of discrete lessons, and inflexible manuals, and cannot be expected to work in a system without appropriate foundations for wellbeing provision. As Green [31] suggests, best practice is a process rather than a packaged intervention: “a common misunderstanding about health promotion research is that it seeks or should seek a magic bullet” (p.173). While high fidelity – the extent to which implementation occurs as intended – is often seen as critical to intervention success, Lendrum and Humphrey [42] suggest that potential for local adaptations may be *beneficial*, enhancing ownership, commitment and goodness-of-fit. Our participants’ emphasis on coherent provision in a setting appears to agree with this theory, and suggests that flexibility may not only improve teachers’ commitment to such provision, but also that of children and young people themselves, where such provision also reflects and speaks to a wider culture embracing wellbeing.

### Social relationships

The quality of emotional and social connections between teachers and students in supporting the emotional and academic needs is well established [43, 44]. However, our analysis also highlights tensions. The multifaceted role of teachers, including as authority figures, was seen to have an undermining effect, and participants perceived inconsistencies in the value placed on wellbeing given that teachers enact systems that may have adverse wellbeing consequences (e.g., current single assessment approaches to high stakes exams). A paradoxical situation has arisen for education settings in supporting wellbeing when wellbeing itself may be adversely affected through traditional behaviourist approaches to classroom management and attainment measures [45]. Our analysis highlights a challenge for teachers: a balance between fostering strong emotional connections and as enforcers of rules and potentially detrimental educational mandates handed down to them. As explored by our participants, such role ambiguity risks leaving children and young people uncertain of what the adults supporting them value. Analysis also pointed to potential inequalities in teacher-student relationships; although power differentials are perhaps inevitable, and indeed at times necessary, relational inequalities could feel at odds with the trust, respect, and kindness emphasised by our participants. Our study adds to growing evidence that elements such as trust and egalitarianism can affect how student-teacher relationships form part of wellbeing provision [16,35,46], and deepens understanding of the complex, multifaceted nature of teachers’ role in a system attempting to balance learning, wellbeing, and classroom management.

Relational aspects of education-based wellbeing support encompass more than teacher-student relationships. Our analysis reflects the value placed on peer support – both formal and informal – and a need to foster friendships and peer interactions. Peer support has long been utilised in education settings to support academic learning, and as a means of supporting wellbeing through initiatives such as befriending, peer mentoring/counselling, and peer mediation strategies [47]. Most research in this area is quantitative or engages with teachers, but some qualitative studies with adolescents have shown positive views of peer support models, including that they can strengthen social community and embed wellbeing values among students (e.g., [48]). Our analysis importantly demonstrates that children and young people spanning age groups and setting types consider peers to play a role that underpins and forms part of wellbeing provision. This could be channeled through formal approaches such as peer mentoring but also more generally supported as an embedded part of daily school life. This can be thought of as complementing staff roles, since our analysis indicates that children and young people see peers as more relatable and indeed other studies show they turn to different sources of support depending on the issue and what they feel they need [15, 49]. However, peer support can be challenging, and there is a need for training and support to facilitate safe, effective approach that give agency while allowing individuals to recognise when support is beyond their means [50]. Moreover, issues of confidentiality and stigma highlighted by participants shows that safety and risk management must be at the forefront [51]. More broadly, opportunities to foster positive peer relationships and friendships were highlighted by participants. Positive relationships with peers – even above relationships with teachers – has been found to be important for school connectedness and belonging [52]. At the forefront, our participants emphasised developing a supportive, kind culture, echoing McGrath and Noble [53], who state that an intentional safe, inclusive, and caring environment is a foundation for positive relationships between both peers and teachers.

### Children and young people at the heart of provision

Children and young people discussed the importance of being active and valued partners, pointing to a need for participatory systems recognising their voices and rights. There is growing emphasis on child and youth voice in system change through consultation and co-design, in line with their right to have a say on matters affecting them [54]. Efforts to engage students in such decisions require meaningful engagement beyond one-off meetings or tokenism [55], though studies indicate that students often report experiencing the latter, with their views ostensibly sought but ultimately ignored in decision-making [35]. Simmons et al.’s study [16] demonstrated that children and young people have much to say on wellbeing provision, and wish for opportunity to do so; our study offers further evidence that they are keen to feed into these decisions.

There is an everyday component here for centring and adapting to children and young people’s needs. It has been argued that a rights-framed approach, with a culture of respect for students’ rights and voices, could be transformative in education, including empowering students in supporting their own wellbeing [56]. Such a framework could encourage flexibility and sensitivity to benefit all, including those with complex needs and circumstances, by making personalisation the norm. There are challenges in embedding rights-framed discourses in education, including conceptual confusion, scepticism and feelings of threat, tokenism, and risk that higher forces could harness ‘rights’ within neoliberal performance agendas [57]. Despite such challenges – and to tackle them – ongoing exploration among policymakers, practitioners, and academics on how settings can incorporate egalitarianism are necessary in efforts to promote wellbeing.

### Vulnerability

Our analysis raises considerations about how wellbeing provision can be designed and implemented with sensitivity to the vulnerabilities it can inadvertently create, with participants pointing to risk of isolation, judgement, and jealousy for those experiencing difficulties and/or engaging with targeted provision. Indeed, even in creating provision in their fictional settings, participants prioritised privacy and confidentiality around wellbeing provision. To *some* extent, a culture that embraces wellbeing and encourages compassion could normalise attending to one’s own and others’ wellbeing and could hopefully reduce, but not eliminate, issues of stigma. In a recent systematic review, Radez et al. [58] noted that the second most commonly reported theme on barriers and facilitators to children and young people seeking and accessing professional mental health help – including in schools – was ‘social factors’, including perceptions of stigma and concerns about embarrassment. Another qualitative systematic review illustrated that, in line with the concerns of our participants, both anticipated and actual experiences of stigma created reluctance and negative consequences around engagement in targeted school-based mental health interventions [59]. Radez et al. [58] suggest that framing provision as positive could reduce negative responses and instead make participants feel ‘lucky’; however, our participants noted risk of jealousy, so this too could have ramifications. Participants emphasised confidentiality but expressed distrust that this would occur, with perceived risks of formal and informal information sharing; other studies have pointed to the value of steps to aid sensitivity and discretion, including clear explanations of privacy and confidentiality [60], referral systems maintaining confidentiality [61], and attending to the physical environment (e.g., avoiding locations in busy spaces, restricting visibility [62]). Underlying such steps is awareness among staff as to how children and young people experiencing difficulties and/or receiving support can feel – and be – vulnerable, and sensitivity to their actions and the social and physical environment.

### Strengths and limitations

This study makes a rigorous, methodologically innovative contribution to evidence on children and young people’s views on education-based wellbeing provision. We highlight our attention to ‘big picture’ considerations beyond discrete domains or interventions; few studies have done this, and similar work from Simmons et al. [16] precedes major policy shifts and the pandemic. A key strength is our focus on children and young people’s views, and indeed our diverse sample means our analysis offers insights from those seldom heard in existing research but often considered vulnerable in terms of wellbeing needs (e.g., those from families with low income). Though we have attended to nuanced aspects of experience in our data (e.g., where perceptions appear particularly to occur among older participants or those in alternative provision) analysis may overlook nuanced aspects of specific views; further work with varying groups across contexts can complement this study. A methodological strength is our consultation with children and young people to design our approach to focus groups; such consultation has rarely been included in work in this area and these discussions critically enhanced our engagement with participants and the meaningfulness of our data. However, future work could build on our design at a deeper level through co-production. We highlight our use of open research practices both for transparency and to guide others planning similar work, making available data generation materials on the Open Science Framework [20], critical reflections on our methods [21], and our analytic codes (Supplementary Materials).

There are considerations around recruitment and data generation occurring via education settings. It is possible that settings who expressed interest in engaging with us were particularly focused on wellbeing, potentially affecting participants’ perceptions. Though we emphasised to staff our wish to engage diverse voices, it is possible some selected students perceived as more likely to ‘behave’ or provide positive views. Finally, though we did our best to mitigate the presence of education staff supporting focus groups, some participants may not have felt able to be direct about aspects of their views, particularly those with less positive experiences; however, we highlight that several participants did appear to talk frankly about challenges, including some directly criticising aspects of their own experiences, and the children and young people who consulted on our design reported that the presence of a school adult could be a reassuring rather than limiting factor.

## Conclusions

As national and international public health policy continues to position wellbeing promotion and prevention as a key role of education settings, and researchers, policymakers, and practitioners explore the complexities of enacting this, it is imperative that we explore children and young people’s perspectives. Our study offers an original and rigorous advancement of knowledge and understanding, presenting a vision from children and young people of an integrated systems approach underpinned by a relational, participatory culture in which wellbeing and students’ needs are prioritised and treated with sensitivity. Our analysis highlights, however, that wellbeing provision in educational domains is not without its challenges, with various tensions risking undermining efforts. This is a critical contribution, showing that tensions well known at policy levels are affecting how children and young people themselves view and engage with wellbeing efforts. We point to a need for critical reflection and ambitious, innovative reforms, if we are to advance policy and provision to promote wellbeing authentically and systematically. There is a critical need to better integrate academic and wellbeing objectives to function as complementary, rather than counterintuitive. There is also a clear value in thinking beyond discrete, prescriptive practices, and instead fostering a caring social community wherein children and young people are supported in forming positive, trusting relationships with educators and one another. This includes exploration of egalitarian and participatory means of engaging with students, particularly but not exclusively in relation to wellbeing promotion and support. Though such reforms are complex and require investment, resourcing, and support for the education workforce, it seems that they are necessary to achieve a vision of wellbeing as part of education in a manner that works for children and young people.

## Electronic supplementary material

Below is the link to the electronic supplementary material.

Supplementary Material 1

## Data Availability

Data cannot be made available due to ethics and privacy restrictions in line with the nature of our consent and assent with our participants and their parents/carers. All data generation materials from the project are publicly available via the Open Science Framework: 10.17605/OSF.IO/R6NVW. We have shared a list of all codes underpinning our themes in the Supplementary Materials of this article.

## Footnotes

1 Our use of the term ‘social, emotional, and mental wellbeing’ is intended to recognise wellbeing as a multifaceted construct encompassing a range of components. For the purposes of this study. we include positive mental health in our use of ‘wellbeing’. Hereafter we refer to this simply as wellbeing unless clarifying specific details of a study or our participants’ wording.

2 The guideline referred to in this article was produced by NICE. We published on main findings for our funder [19] where we focused more narrowly on specific practices and strategies to inform new NICE guidelines for education-based wellbeing provision; here we instead look more broadly to understand how this sits within the wider school context.

3 In England, alternative provision settings provide education to children and young people who are not able to attend a mainstream school, often (though not exclusively) due to permanent or fixed-period exclusions.

4 We report on the broadest category of ethnicity here to preserve anonymity of individual children and young people from specific ethnic groups less proportionally represented in the wider sample.

5 We note that this may seem to be in opposition to points in a previous theme about creating space for sharing feelings; to some extent this contradiction was present within the data, though there were also considerations put forward such as ensuring that children and young people not being required to talk about their own personal emotions in these conversations. Ultimately these contradictions highlight the complexity of facilitating aspects of wellbeing provisions in ways that can best support the ranging needs of children and young people.

## List of abbreviations

ADHD: Attention deficit hyperactivity disorder
EAL: English as an additional language
FSM: Free school meals
KS: Key stage
NICE: The National Institute for Health and Care Excellence
SEND: Special educational needs and disability
OSF: The Open Science Framework
