# Supplementary material for: “We want it to be a culture”: children and young people’s perceptions of what underpins and undermines education-based wellbeing provision
Source: BMC Public Health. 2023 Jul 7;23:1305. doi: 10.1186/s12889-023-15836-z (PMC10327321; doi:10.1186/s12889-023-15836-z)
Supplement: Supplementary file 1 — Supplementary Material 1 [file 12889_2023_15836_MOESM1_ESM.docx]

**Supplementary Materials: Codes Underpinning Themes**

| Themes and nested codes | Transcripts  (7 total) | References |
| --- | --- | --- |
| **1) Recognising and facilitating the setting as a caring social community** | 7 | 81 |
| A space to learn social skills | 3 | 5 |
| Helping children learn to be social | 1 | 1 |
| Learning social skills | 1 | 1 |
| Preparing for real life and social skills | 1 | 1 |
| Teach people social skills | 1 | 1 |
| Teaching about social relationships | 1 | 1 |
| Compassion and kindness | 7 | 53 |
| Addressing worries for new students | 1 | 1 |
| Appreciating kindness and compassion makes school a better place | 1 | 1 |
| Be kind and respectful | 1 | 3 |
| Being flexible, not judging people | 1 | 1 |
| Being friendly | 1 | 1 |
| Being helpful and kind is critical | 1 | 1 |
| Being kind | 1 | 1 |
| Being kind (2) | 1 | 1 |
| Being welcoming and friendly | 1 | 1 |
| Challenging stigma and poor behaviour around MHW | 1 | 1 |
| Feeling safe and happy | 1 | 1 |
| Focus on treating people around you well | 1 | 1 |
| Give children encouragement | 1 | 1 |
| Giving comfort and reassurance | 1 | 1 |
| Happy and kind as value | 1 | 1 |
| Having values is important | 1 | 1 |
| Help children feel comfortable (teachers) | 1 | 2 |
| Help make new students feel less nervous | 1 | 1 |
| Helping people feel comfortable | 1 | 1 |
| Helping people feel comfortable | 1 | 1 |
| If everyone is mean everyone would leave | 1 | 1 |
| If you're stressed you don't want to come to school | 1 | 1 |
| Kind to others | 1 | 4 |
| People in schools should be nice | 1 | 1 |
| Positive environment | 1 | 1 |
| Positivity in school | 1 | 1 |
| Saving YP stress and worry and dread | 1 | 1 |
| School could have been lava, deep end after COVID | 1 | 1 |
| School has responsibility for wellbeing | 1 | 1 |
| School values can help reduce bullying | 1 | 1 |
| Schools are supposed to promote kindness | 1 | 1 |
| Sharing values on social support | 1 | 2 |
| So people are not horrible to others | 1 | 1 |
| Social and mental wellbeing seen as interrelated | 1 | 2 |
| Social values more important than rules | 1 | 1 |
| Teach about being mean vs kind | 1 | 1 |
| Teachers boosting students | 1 | 1 |
| Teachers CAN be controlled, not pupils | 1 | 1 |
| Teaching children to be kind | 1 | 2 |
| Teaching how to be kind and calm | 1 | 1 |
| Tell people how to be kind | 1 | 1 |
| There needs to be social cohesion in a school | 1 | 1 |
| Vital importance of being nice | 1 | 1 |
| Wanting people to get along | 1 | 1 |
| Social connection in school (especially for those struggling) | 6 | 23 |
| Connecting with other pupils | 1 | 1 |
| Create opportunities to connect | 1 | 1 |
| Creating final memories together | 1 | 1 |
| Emphasis on social connection | 1 | 2 |
| Feeling connected | 1 | 1 |
| Helping children get along better | 1 | 2 |
| Helping create friendships to support wellbeing | 1 | 1 |
| Helping people make friends and not be lonely | 1 | 1 |
| Helping someone make friends = making someone happier | 1 | 2 |
| Important to help social relationships and stop bullying | 1 | 1 |
| Important to help someone make friends | 1 | 1 |
| Lessons don't replace experience | 1 | 1 |
| Letting people reconnect post-COVID | 1 | 1 |
| Make people feel special, valued | 1 | 2 |
| Making sure people don't feel alone | 1 | 1 |
| Need to build connections with staff | 1 | 1 |
| Personal contact with teachers | 1 | 1 |
| Supporting social inclusion and friendships | 1 | 1 |
| There needs to be social cohesion in a school | 1 | 1 |
| **2) Enabling wellbeing to be a central setting priority** | 7 | 119 |
| Everyone is responsible for culture | 5 | 10 |
| Epic divide if teachers don't follow | 1 | 1 |
| Everybody has to buy in | 1 | 1 |
| Everyone is responsible for culture | 1 | 1 |
| Everyone should feel equal | 1 | 1 |
| EVERYONE should have a say | 1 | 1 |
| Everyone should know the rules | 1 | 1 |
| Fairness | 1 | 1 |
| Values are for everyone | 1 | 1 |
| Values should be equal across students and teachers | 1 | 1 |
| Values should be the same for everyone | 1 | 1 |
| Genuinely embedded culture of wellbeing, including normalisation | 7 | 36 |
| Can provision help everyone | 1 | 1 |
| Culture not rules | 1 | 1 |
| Do people really think values are important in practice | 1 | 1 |
| Embedding mental health across the curriculum | 1 | 1 |
| Embedding talking | 1 | 1 |
| Embedding values | 1 | 1 |
| Embedding values, not forcing rules | 1 | 1 |
| Extending delivery of mental health rather than one-offs | 1 | 3 |
| If school is for calming then it should calm you down | 1 | 1 |
| Important to understand how values affect you | 1 | 1 |
| Make sure people know the school values | 1 | 1 |
| Making sure people with difficulties don't feel alone | 1 | 1 |
| Mental health central to school identity | 1 | 2 |
| Mental health is a priority thing to learn | 1 | 1 |
| Needs to be taught regularly | 1 | 1 |
| Normalising it's okay not to be okay | 1 | 1 |
| Prioritising different values in a school | 1 | 1 |
| Repeating and building understanding | 1 | 2 |
| School and class level communication and discussion | 1 | 2 |
| Shared values are important | 1 | 3 |
| Shouldn't be for the sake of it | 1 | 1 |
| Student body as role models | 1 | 2 |
| Support does not need to be limited to a lesson | 1 | 1 |
| Support needs to be consistent | 1 | 1 |
| Sustained engagement | 1 | 1 |
| Talking and normalising would improve help seeking | 1 | 1 |
| Universal classes help operationalise school culture | 1 | 1 |
| Will CYP actually care about school values | 1 | 1 |
| School as a site for wellbeing promotion and support | 7 | 41 |
| CYP might not talk to parents and friends about MHW, school can create this space | 1 | 1 |
| Ensuring there is MHW support in transition | 1 | 1 |
| General preference for teacher | 1 | 1 |
| Good to normalise mental health | 1 | 1 |
| Government is in charge of school | 1 | 1 |
| Help us understand our mental health | 1 | 1 |
| Helping people prepare for transition | 1 | 1 |
| Mental health people coming to school | 1 | 1 |
| School should help people feel happy | 1 | 3 |
| School staff are the people who should help | 1 | 1 |
| Schools are supposed to promote kindness | 1 | 1 |
| Schools can help you | 1 | 1 |
| Schools have a responsibility when transition is hard | 1 | 1 |
| Schools need mental health funding | 1 | 1 |
| Specialist mental health knowledge and experience | 1 | 1 |
| Staff influencing wellbeing culture | 4 | 8 |
| SLT and teachers deciding on school values | 1 | 1 |
| Staff might need a different set of values | 1 | 1 |
| Staff should have a say, they are doing the work | 1 | 1 |
| Teachers are well equipped to make these decisions | 1 | 1 |
| Teachers give reassurance | 1 | 1 |
| Teachers have impact for culture | 1 | 1 |
| Teachers should also carry values | 1 | 2 |
| Teachers are a sensible route of support in theory | 1 | 1 |
| Teachers as helpers | 1 | 1 |
| Teachers can help | 1 | 1 |
| Teachers can help children | 0 | 0 |
| Teachers can make things easier | 1 | 2 |
| Teachers have some responsibility | 1 | 1 |
| Teachers there to teach AND look after you | 1 | 1 |
| The onus should be on teachers | 1 | 1 |
| The teachers could help support skill development | 1 | 1 |
| Time in lessons to talk about mental health | 1 | 1 |
| Wellbeing provision and intervention to avoid worsening of difficulties | 4 | 6 |
| Avoiding later consequences | 1 | 1 |
| Avoiding worse consequences, depression | 1 | 1 |
| Helping children is important to prevent later consequences | 1 | 2 |
| Preventing other consequences like suicide | 1 | 1 |
| Things could get worse without help | 1 | 1 |
| School has demands and stress | 3 | 9 |
| Avoid unnecessary strain for students (and staff) | 1 | 1 |
| Avoiding unnecessary stress | 1 | 1 |
| Balancing demands on young people | 1 | 1 |
| Classroom is stressful | 1 | 1 |
| Lessons in managing exams, homework, to help with MHW | 1 | 1 |
| Pressure to 'catch up' after COVID - anxiety and stress | 1 | 1 |
| School causes difficulties for mental health | 1 | 2 |
| The classroom can be irritating | 1 | 1 |
| The 'real' function of school and teachers | 6 | 23 |
| Blurred lines in teacher role | 1 | 1 |
| Entire education system would need to change | 1 | 5 |
| Flexibility could mean missing education | 1 | 1 |
| Headteacher too busy for this | 1 | 1 |
| How much can schools really do | 1 | 2 |
| Is mental health a teacher's job | 1 | 2 |
| Limit wellbeing support to prioritise learning | 1 | 1 |
| Making sure they don't miss their learning getting support | 1 | 2 |
| Mind your business | 1 | 1 |
| School isn’t in control of priorities | 1 | 1 |
| School as a place where you do solid work the whole time | 1 | 1 |
| TAs should do it so teachers can plan | 1 | 1 |
| Teachers are getting paid | 1 | 1 |
| Wellbeing support competes with learning | 1 | 2 |
| Wellbeing team's job | 1 | 1 |
| **3) Facilitating strong relationships with staff who understand and care about wellbeing** | 7 | 144 |
| A warm teaching persona | 6 | 20 |
| A 'whole' relationship | 1 | 1 |
| A certain teacher | 1 | 1 |
| A favourite teacher you trust can talk to you | 1 | 1 |
| An existing relationship helps | 1 | 1 |
| Be relaxed with students | 1 | 1 |
| Build good relationships with pupils | 1 | 1 |
| Building genuine teacher relationships | 1 | 1 |
| It needs to be a teacher that teaches you | 1 | 1 |
| It should be a favourite teacher | 1 | 1 |
| Pastoral might be removed from children YP | 1 | 1 |
| People who know you and your needs | 1 | 1 |
| Relational dynamics that help | 0 | 0 |
| A need for discussions to feel safe and comfortable | 1 | 1 |
| Comfort trumps training | 1 | 1 |
| Feeling comfortable with a teacher | 1 | 1 |
| Finding teachers you feel comfortable with | 1 | 1 |
| Importance of trust | 1 | 1 |
| Mutual respect between pupils and teachers | 1 | 1 |
| Need to feel comfortable to ask for help | 1 | 1 |
| Someone you feel comfortable around | 1 | 1 |
| Stop being strict all the time | 1 | 1 |
| Teachers being friendly | 1 | 1 |
| Teachers need to be approachable | 1 | 2 |
| Teachers need to be genuinely interested in you | 0 | 0 |
| Teachers should be kind | 1 | 1 |
| Teachers should be nice and show an interest | 1 | 1 |
| Teachers should be real people | 1 | 2 |
| Teachers should know about your problems so they can help better | 1 | 1 |
| Teachers should respect you | 1 | 2 |
| Teachers should teach because they enjoy it, not for money | 1 | 1 |
| Teachers showing flexibility | 1 | 1 |
| There has to be rules, but things should be flexible | 1 | 1 |
| Things should feel human, not awkward | 1 | 1 |
| Wanting a teacher that can relate to you | 1 | 1 |
| Wanting people who have experience | 1 | 1 |
| Wanting someone who knows everything about you already | 1 | 1 |
| Relationship with teachers affects engagement with wellbeing sessions | 1 | 1 |
| Relationship with your teacher matters | 1 | 1 |
| Seeing high school teachers as super serious | 1 | 1 |
| Support should be from someone who is familiar and has a relationship with you | 1 | 1 |
| Teacher can be a friend | 1 | 1 |
| Teachers already know your context | 1 | 1 |
| Teachers should be your friend | 1 | 1 |
| Wanting someone who knows everything about you already | 1 | 1 |
| You don't tell a 'randomer' things | 1 | 1 |
| Considering the needs of staff, too (including training) | 6 | 18 |
| A teacher's job is very stressful | 1 | 1 |
| Avoid unnecessary strain for students (and staff) | 1 | 1 |
| Government should help out teachers | 1 | 1 |
| Mental health training | 1 | 1 |
| More training for teachers needs money and government support | 1 | 1 |
| Someone who is knowledgeable | 1 | 1 |
| Someone who is trained to handle mental health | 1 | 1 |
| Teachers are familiar and could be safer if trained | 1 | 1 |
| Teachers are qualified to teach us things | 1 | 1 |
| Teachers in a balancing act | 1 | 1 |
| Teachers might not understand mental health | 1 | 1 |
| Teachers need more training | 1 | 1 |
| Teachers need to know the signs | 1 | 1 |
| Teachers need training | 1 | 2 |
| Teachers need training to look for signs | 1 | 1 |
| Teachers should be trained to know how to be helpful | 1 | 1 |
| Thinking about teachers' needs too | 1 | 1 |
| Feeling like you cannot talk to a teacher | 4 | 26 |
| Bad experiences with teachers | 1 | 1 |
| Class teacher might not always be the best person | 1 | 1 |
| I don't go to a teacher | 1 | 1 |
| Initial hesitation about wellbeing team | 1 | 1 |
| It can be intimidating to go and talk to teachers | 1 | 1 |
| It's embarrassing to ask for help at school | 1 | 1 |
| It's hard to ask for help at school | 1 | 1 |
| Mind your business | 1 | 1 |
| Nobody asks teachers for help | 1 | 1 |
| People don't actually talk to teachers | 1 | 2 |
| People don't always want to talk to teachers | 1 | 1 |
| Teacher might not relate | 1 | 1 |
| Teachers are a pest | 0 | 0 |
| Teachers are too busy | 1 | 1 |
| Teachers don't really care | 1 | 2 |
| Teachers might not be honest with you | 1 | 1 |
| Teachers might not relate to us | 1 | 1 |
| Teachers might not relate to young people | 1 | 2 |
| Teachers might not understand | 1 | 1 |
| Teachers might say unhelpful things | 1 | 1 |
| Teachers take things the wrong way | 1 | 1 |
| Trust kids more than adults | 1 | 1 |
| Wanting someone who is not a teacher | 1 | 1 |
| Wellbeing team not always the best fit | 1 | 1 |
| Feeling you can ask for help and share how you feel (including clear routes) | 6 | 38 |
| Being able to ask for help | 1 | 1 |
| Being able to tell teachers how you feel | 1 | 1 |
| Children knowing they can speak to anyone any time | 1 | 1 |
| Create space to talk about worries | 1 | 1 |
| Creating opportunities to talk | 1 | 2 |
| Creating safe environment by including wellbeing talk and improving communication | 1 | 1 |
| creating ways to let out feelings | 1 | 1 |
| Encouraging CYP to ask for help when they are not happy | 1 | 1 |
| Ensuring comfortable space to talk | 1 | 1 |
| Good to normalise mental health | 1 | 1 |
| Having ways to let people express difficult or embarrassing things | 1 | 1 |
| Helping children get feelings out | 1 | 1 |
| Improving MH communication | 1 | 1 |
| Knowing routes for MHW help | 1 | 1 |
| Knowing who to go to | 1 | 1 |
| Making enough time and space for wellbeing conversations | 1 | 1 |
| Need to communicate feelings easily | 1 | 1 |
| People who deal with problems in school | 1 | 1 |
| Pupils should ask for help | 1 | 2 |
| Safety around talking | 4 | 6 |
| Classroom could be too noisy and difficult | 1 | 1 |
| Classroom is not always the safest space to talk | 1 | 1 |
| Classroom is stressful | 1 | 1 |
| Helping children feel safe | 1 | 1 |
| Mismatch between classroom and sharing feelings | 1 | 1 |
| Needing it to feel relaxed | 1 | 1 |
| Someone having specific responsibility for wellbeing | 1 | 2 |
| Talk about feelings | 1 | 1 |
| Talk to your teachers | 1 | 1 |
| Talking about feelings should be allowed all the time | 1 | 1 |
| Talking and normalising would improve help seeking | 1 | 1 |
| Teachers as someone to tell problems to | 1 | 1 |
| Telling the teacher as a cry for help | 1 | 1 |
| Time to talk to your teacher to calm down | 1 | 1 |
| Understanding ways of sharing | 1 | 1 |
| Want school to be caring, a space you can talk about feelings | 1 | 1 |
| Power and hierarchy issues in school | 5 | 13 |
| Adults tell you what to do | 1 | 1 |
| Authority should not matter in values | 1 | 1 |
| Children and adults should know the value rules | 1 | 1 |
| Discipline is not always the answer | 1 | 1 |
| Epic divide if teachers don't follow | 1 | 1 |
| Feeling told off all the time | 1 | 1 |
| People in the school being equal | 1 | 3 |
| Stop being strict all the time | 0 | 0 |
| Stop being strict all the time (2) | 1 | 1 |
| Teachers enforce unhelpful power dynamic | 1 | 1 |
| Values are for students not teachers | 1 | 1 |
| You're a kid, they're an adult | 1 | 1 |
| Staff should 'know' you and check in | 6 | 29 |
| A student or teacher should see if you're okay | 1 | 1 |
| Asking children what is wrong | 1 | 1 |
| Check in and see how children are doing | 1 | 1 |
| Checking in every day | 1 | 1 |
| Checking in gently | 1 | 1 |
| Creating opportunities to check in and ask for help | 1 | 1 |
| Disruptive behaviour might indicate need | 1 | 1 |
| Find out how people are feeling | 1 | 1 |
| Keep an eye on students | 1 | 1 |
| Look for signs | 1 | 1 |
| Look for signs of difficulties | 1 | 1 |
| Looking 'behind' behaviour | 1 | 1 |
| Looking for signs | 1 | 1 |
| Monitoring behaviour | 1 | 1 |
| Monitoring children and knowing their needs | 1 | 5 |
| Monitoring children's behaviour to see if they need help | 1 | 1 |
| Monitoring needs and issues | 1 | 1 |
| Naughty or angry behaviour tells you they're not okay | 1 | 1 |
| Noting when people aren't themselves | 1 | 1 |
| People who know you and your needs | 1 | 1 |
| Screening and monitoring people | 1 | 1 |
| Teachers creating conversations if people are not happy | 1 | 1 |
| Teachers having mental health conversations | 1 | 1 |
| Teachers should have a quiet word | 1 | 1 |
| Understanding how students feel | 1 | 1 |
| **4) Engaging children and young people as active partners** | 7 | 71 |
| Peers and friends can support each other | 7 | 47 |
| A friend can help you | 1 | 1 |
| A student or teacher should see if you're okay | 1 | 1 |
| Children can help and support each other | 1 | 1 |
| Children can help each other | 1 | 1 |
| Children helping each other | 1 | 4 |
| Creating opportunities to chat and check in with each other | 1 | 1 |
| Creating peer support opportunities and systems | 1 | 1 |
| Creating space to talk | 1 | 1 |
| Creating space to talk to each other | 1 | 5 |
| Creating supported small group discussions | 1 | 1 |
| Friend, peer support | 1 | 1 |
| Helping each other | 1 | 1 |
| Involving peers and friends | 1 | 1 |
| Involving students as peer support | 1 | 3 |
| Involving students in support | 1 | 2 |
| Learning how to support each other | 1 | 1 |
| Needing space to talk to make things better | 1 | 1 |
| Opportunity to talk in small groups | 1 | 1 |
| Peer support | 1 | 1 |
| Peers easier to talk to and relate easier | 5 | 11 |
| Can be easier to speak to other children | 1 | 1 |
| Friends relate better than teachers | 1 | 1 |
| Kids understand and think like you | 1 | 1 |
| Needing someone to relate to you | 1 | 1 |
| Other students are going through the same thing | 1 | 1 |
| Others understanding how you are feeling | 1 | 1 |
| Peers relate better | 1 | 1 |
| Relatability in student body | 1 | 1 |
| Relatedness and empathy | 1 | 1 |
| Students will listen to each other more than teachers | 1 | 1 |
| Teachers don't understand like kids do | 1 | 1 |
| Peers, friends as key support, not just teachers | 1 | 1 |
| Small group discussions | 1 | 1 |
| Small group talking, not whole class | 1 | 1 |
| Space to talk about whatever | 1 | 1 |
| Space to voice concerns and help each other | 1 | 1 |
| Time to talk to each other | 1 | 1 |
| Trust kids more than adults | 1 | 1 |
| Pupil voice and choice | 5 | 24 |
| A wide range of voices feeding into plans | 1 | 1 |
| All students should have a chance to feed in | 1 | 1 |
| Be inclusive of pupils | 1 | 2 |
| Children making decisions | 1 | 1 |
| Creating choice | 1 | 1 |
| CYP being part of decisions about values | 1 | 1 |
| Don't force discussion | 1 | 1 |
| Ensure a variety of pupil voice | 1 | 1 |
| Focus more on student voice than teachers | 1 | 1 |
| Get young people involved in decisions about them | 1 | 1 |
| Helping children understand what is available to them e.g. a room if they feel anxious | 1 | 1 |
| If students make values they will uphold them | 1 | 1 |
| Importance of pupil choice | 1 | 1 |
| Kids should help decide because they know what gets on their nerves | 1 | 1 |
| Make people feel like they're involved in setting and exploring the values | 1 | 1 |
| Mutual agreements and middle grounds | 1 | 1 |
| Pupil voice and needs | 1 | 1 |
| Pupils are part of progression of school | 1 | 1 |
| Pupils should feel like they have a say | 1 | 1 |
| Student buy in | 1 | 2 |
| Students got to have a say | 1 | 1 |
| Surveying students for their input | 1 | 1 |
| **5) Adapting to collective and individual needs** | 7 | 68 |
| Adapting and tailoring for individual CYP needs | 7 | 42 |
| Adapting to people's needs and circumstances | 1 | 1 |
| Adjusting around students' individual needs | 1 | 2 |
| Allowing CYP to have a break | 1 | 1 |
| Allowing time out | 1 | 1 |
| Being aware of individual needs | 1 | 1 |
| Being fair | 1 | 1 |
| Being flexible to individuals | 1 | 2 |
| Considering how things can be personalised to students rather than en masse | 1 | 1 |
| Creating time away when needed | 1 | 1 |
| Discipline is not always the answer | 1 | 1 |
| Does everyone need the same thing in a universal session | 1 | 1 |
| Genuinely seeing students as individuals with individual needs | 1 | 2 |
| Don't label or dismiss student needs | 1 | 1 |
| Don't lump students together | 1 | 1 |
| Give student space | 1 | 1 |
| Giving extra help when needed | 1 | 3 |
| Help support people who are nervous | 1 | 1 |
| Helping children calm down with things they like e.g., music | 1 | 1 |
| Helping those who need it | 1 | 1 |
| Identifying barriers and helping | 1 | 1 |
| Individual YP needs and wants | 1 | 1 |
| Knowing individual children's needs | 1 | 1 |
| Levels of support universal v. 1.1 support | 1 | 1 |
| Make sure things don't seem unfair | 1 | 2 |
| Might need support at some stages not others | 1 | 1 |
| Need for multiple provision if you need desperate help | 1 | 1 |
| Need to tailor to YP needs | 1 | 1 |
| Offering support tailored to needs | 1 | 1 |
| Respond to individual needs | 1 | 1 |
| Self-care without getting in trouble | 1 | 1 |
| Some kids need more help, we should give it | 1 | 1 |
| Support should be tailored | 1 | 1 |
| Teachers being sensitive to children's needs | 1 | 1 |
| Teachers should know about your problems so they can help better | 1 | 1 |
| Teachers showing flexibility | 1 | 1 |
| Understanding differentiated need | 1 | 1 |
| Understanding individual needs | 1 | 1 |
| YP should have flexibility in school day | 1 | 1 |
| Provision that considers CYP needs | 6 | 26 |
| Allowing some flexibility | 1 | 1 |
| Anticipating needs of new students | 1 | 1 |
| Does everyone need the same thing in a universal session | 1 | 1 |
| Ease back in slowly after COVID | 1 | 1 |
| Easing people into things | 1 | 1 |
| Flexibility after COVID | 1 | 2 |
| Getting comfortable in the classroom again | 1 | 1 |
| Help children feel comfortable (teachers) | 1 | 2 |
| Help make new students feel less nervous | 1 | 1 |
| Helping people feel comfortable | 1 | 1 |
| Helping people feel comfortable | 1 | 1 |
| Importance of communicating expectations for transition | 1 | 1 |
| Laying off a bit after COVID | 1 | 1 |
| Make sure pupils feel safe | 1 | 1 |
| Return to 'normal' slowly after COVID | 1 | 2 |
| Saving YP stress and worry and dread | 1 | 1 |
| Staggered age-appropriate learning | 1 | 1 |
| Teaching skills applicable to everybody | 1 | 1 |
| Understanding children's needs after COVID | 1 | 2 |
| Work not the priority after COVID | 1 | 3 |
| **6) Being discreet and sensitive to vulnerability** | 6 | 50 |
| Discretion and confidentiality | 5 | 22 |
| A need for privacy and discretion | 1 | 1 |
| Accessing help isn't confidential - this is off-putting | 1 | 1 |
| Being discreet | 1 | 1 |
| Being discreet and creating space for private talk | 1 | 1 |
| Being discreet to avoid embarrassment | 1 | 3 |
| Confidentiality might be broken | 1 | 1 |
| Discretion | 1 | 1 |
| Discretion (2) | 1 | 2 |
| Freedom to speak without fear of information sharing | 1 | 1 |
| Issues around confidentiality and information sharing | 1 | 1 |
| Needing privacy in getting support | 1 | 1 |
| Private conversations | 1 | 2 |
| Teacher sharing it would breach boundary | 1 | 1 |
| Teachers might share what we say | 1 | 2 |
| Teachers should have a quiet word | 1 | 1 |
| Trust kids more than adults | 1 | 1 |
| Wanting privacy and confidentiality | 1 | 1 |
| Vulnerability and wellbeing in school | 5 | 28 |
| Avoid making people vulnerable | 1 | 1 |
| Avoiding unintended consequences | 1 | 1 |
| Avoiding unintended consequences (2) | 1 | 1 |
| Avoiding unintended consequences (3) | 1 | 1 |
| Being careful in MH conversations with younger children | 1 | 1 |
| Care when teaching vulnerable ages | 1 | 1 |
| Challenging stigma and poor behaviour around MHW | 1 | 1 |
| Challenging stigma and poor behaviour around MHW (2) | 1 | 1 |
| Children might get jealous of flexibility | 1 | 1 |
| Could do more damage than good | 1 | 1 |
| Counselling and stigma | 1 | 1 |
| Ensure people are not excluded | 1 | 1 |
| Feeling forced to share | 1 | 1 |
| Help children understand different consequences | 1 | 1 |
| How much can teachers do | 1 | 1 |
| Isolation of pupils with difficulties | 1 | 1 |
| Making sure students are not 'the odd one out' if they have MH problems | 1 | 1 |
| Other people judging you for needing help | 1 | 1 |
| Other pupils responding negatively to needs | 1 | 1 |
| People might be jealous about help | 1 | 1 |
| Pupils may mock you for having difficulties | 1 | 2 |
| Risk that safe spaces become stigmatised | 1 | 2 |
| Scaling so not triggering | 1 | 1 |
| Talking about MH could make some people feel different, isolated | 1 | 1 |
| Talking about MHW in a class is vulnerable | 1 | 1 |
| Talking MH is still vulnerable for young people in this day and age | 1 | 1 |
| Additional codes later discarded as judged to be thin, sporadic, or unrelated to research question | 7 | 48 |
| Make it interesting | 3 | 5 |
| Creating discussion and interaction | 1 | 1 |
| Don't do too much talking with everyone | 1 | 1 |
| Interactive approaches | 1 | 1 |
| It might be boring | 1 | 1 |
| Should be challenging | 1 | 1 |
| Preparing for adulthood | 4 | 15 |
| Focusing on progression in life | 1 | 1 |
| Help prepare students for their next steps e.g., university | 1 | 1 |
| Help set expectations about next steps | 1 | 1 |
| Helping people prepare to be citizen | 1 | 4 |
| Keeping people on the straight and narrow | 1 | 1 |
| Knowing what's right and wrong, having rules | 1 | 1 |
| School has rules that aren't like 'real life' | 1 | 1 |
| School isn't the same as real life | 1 | 1 |
| School prepares you to be a good citizen | 1 | 2 |
| Wanting to be taught life skills | 1 | 2 |
| BUT values are longstanding - does everyone buy in | 1 | 1 |
| Communicating values | 1 | 2 |
| Creating a safe learning environment through lifelong learning | 1 | 1 |
| Creating distraction and room away from difficulties | 1 | 1 |
| Creating relaxation (art, mindfulness) | 1 | 4 |
| Encouraging positive thinking | 1 | 1 |
| Having values that are 'real life' | 1 | 1 |
| Helping people get over difficult feelings and situations | 1 | 1 |
| Helping people manage feelings | 1 | 1 |
| Hidden curriculum rather than taught | 1 | 1 |
| Promoting wellbeing includes changing the school | 1 | 1 |
| Responding to stress | 1 | 1 |
| School requires some order | 1 | 1 |
| School tries to make things fair | 1 | 1 |
| Space to voice worries | 1 | 1 |
| Talking about feelings helps make you calm | 1 | 1 |
| The role of parents | 4 | 6 |
| Involving parents | 1 | 1 |
| Involving parents and families | 1 | 3 |
| Parents have ownership | 1 | 1 |
| Parents should have a say in culture | 1 | 1 |
| Things that help with feelings (e.g., mindfulness) | 1 | 1 |
| Time in lessons to relax | 1 | 1 |
